# Implementing psychedelic-assisted therapy: History and characteristics of the Swiss limited medical use program

**DOI:** 10.1016/j.nsa.2025.105525

**Published:** 2025-07-31

**Authors:** Matthias E. Liechti, Peter Gasser, Helena D. Aicher, Felix Mueller, Tadeusz Hawrot, Yasmin Schmid

**Affiliations:** a Division of Clinical Pharmacology and Toxicology, Department of Clinical Research, University Hospital Basel and University of Basel, Basel, Switzerland; b Department of Pharmaceutical Sciences, University of Basel, Basel, Switzerland; c Practice for Psychiatry and Psychotherapy, Solothurn, Switzerland; d Swiss Medical Association for Psychedelic Therapy (SÄPT), Switzerland; e Department of Psychiatry, University of Basel, Basel, Switzerland; f Psychedelic Access and Research European Alliance (PAREA), Brussels, Belgium; g European Federation of Neurological Associations, Brussels, Belgium

**Keywords:** LSD, Psilocybin, MDMA, Psychedelic, Limited access, Medical use

## Abstract

This article describes the Swiss limited access program for psychedelic/3,4-methylenedioxymethamphetamine (MDMA)-assisted therapy. The Swiss Federal Office of Public Health can issue authorizations for the limited medical use of otherwise prohibited substances. To be eligible, patients suffer from a mostly incurable disease, the prohibited substance can alleviate the suffering, and there are no alternative treatments, or such treatments have already extensively been used with insufficient outcome. The current program started in 2014 with two physicians. In 2024, there were approximately 100 physicians who held authorizations to treat 723 patients with MDMA (245 patients), lysergic acid diethylamide (130 patients), or psilocybin (348 patients). There were approximately 1660 psychedelic/MDMA-assisted treatments in 2024, with patients typically being treated 2–4 times with the respective substance within 12 months. Various aspects of the program, including its history, provider characteristics and setting, legal requirements, treatment cost, the role of professional societies, education and continuous formation, personal experience, patient characteristics, outcome, and adverse effects, are described and discussed relative to other recently established programs in Canada and Australia. Such information could be of interest to psychedelic-assisted therapy stakeholders, including professionals, patients, and regulatory bodies that are considering setting up similar restricted access programs.

## 1 Introduction

Psychoactive substances, including classic psychedelics (e.g., psilocybin and lysergic acid diethylamide [LSD]) and the empathogen 3,4-methylenedioxymethamphetamine (MDMA), are investigated as medications for psychiatric and other disorders. Currently, psilocybin, LSD, and MDMA are in Phase 3 clinical trials for the treatment of major depressive disorder (MDD), generalized anxiety disorder (GAD), and posttraumatic stress disorder (PTSD), respectively. Market approval for these treatments for primary indications could be expected in 2028 in the United States but remains unclear in Europe. In the meantime and alternatively, special access programs (SAPs) for these novel treatment options have been established in a few countries, including Canada, Australia, and Switzerland (Aicher et al., 2024b; EMA, 2024). Here, we describe characteristics of the Swiss limited use program, which was established over the past 10 years.

## 2 Special access programs

Generally, an SAP can provide exceptional access to substances without the approval of these substances for market access. With regard to psychedelics and MDMA, there are currently three such programs worldwide.

Psilocybin and MDMA were added to Health Canada's SAP in 2022. The program provides healthcare professionals with access to drugs that are not approved to be marketed in Canada but may be needed for the treatment of serious disorders when other treatment options have failed. Currently, psilocybin and MDMA can be obtained. Psilocybin has mainly been used in patients with MDD but also for the treatment of end-of-life psychological distress. MDMA is used in patients with PTSD (EMA, 2024). In 2022–2025, a few hundred patients were treated within this program (EMA, 2024).

Australia reclassified psilocybin and MDMA from prohibited substances to controlled drugs for medical use. Since 2023, psilocybin and MDMA can be used by psychiatrists in patients with treatment-resistant depression (TRD) and PTSD, respectively, through an authorized prescriber pathway. The first patients within this program were treated in 2024 (EMA, 2024).

The current Swiss SAP started in 2014 and is described herein.

## 3 The Swiss limited medical use program

### 3.1 History of psychedelic-assisted therapy (PAT) in Switzerland

**Fig. 1:**
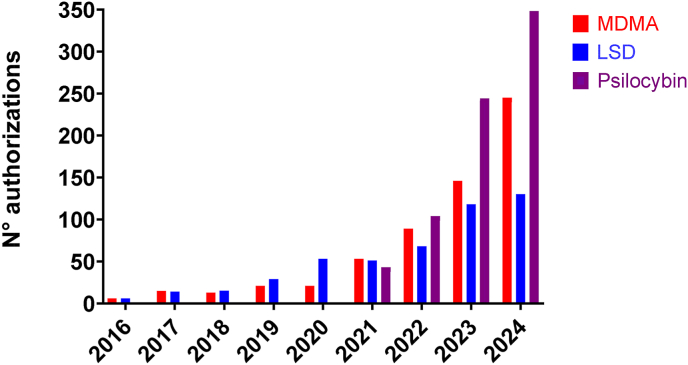
**Number of patients in the Swiss limited use program.** Data represent the number of individual patient treatment authorizations valid for 12 months issued by the Swiss Office of Public Health. Authorizations include novel approvals and extensions granted within the year indicated. A few authorizations did not result in treatment. In a few cases, two authorizations for different substances were issued consecutively for one patient within the same year. Only very few (<5) patients were treated with MDMA or LSD in 2014–2015 by two psychiatrists. Thereafter, the number of patients increased. MDMA and LSD became available in 2014. Psilocybin became available in 2021. Authorizations are valid for 12 months (extension possible). Most patients are treated 2–3 times per year with the respective substance.

In Switzerland, LSD and MDMA were already used by five therapists in the first restricted use program from 1988 to 1993 in patients with different psychiatric disorders (Gasser, 1994, 1996). The current limited medical use program that uses MDMA and LSD started in 2014 upon completion of the first clinical studies using MDMA and LSD in Switzerland, providing minimal evidence of efficacy for PTSD (Oehen et al., 2013) and anxiety in patients with severe illness (Gasser et al., 2014, 2015), respectively. As of 2014, the program provided access to MDMA and LSD. Access to psilocybin was only added in 2021. The number of patients who were treated with each substance is shown in Fig. 1. The number of treated patients strongly increased after 2021. In 2024, a total of 723 treatment authorizations (245 for MDMA, 130 for LSD, and 348 for psilocybin) were issued by the Federal Office of Public Health (FOPH). Almost all authorizations resulted in the treatment of patients with the respective substance at least once and typically several times. There were approximately 1660 psychedelic/MDMA-assisted treatments in 2024, with patients typically treated 2–4 times with the respective substance within the treatment authorization period, which covers 12 months. The number of authorized physicians who were responsible for the treatment of patients increased over the years to approximately 100 in 2024.

### 3.2 Psychedelic-assisted therapy provider and setting

In 2024, there were approximately 80 private practices and 15 institutions, including four university hospitals, that offered PAT in Switzerland. The median number of treated patients per physician was only two (mean = 7 patients, range = 1–72 patients). Notably, a responsible physician, particularly at a larger clinic, can delegate the treatment or parts of the treatment to other physicians in their clinic or practice but also to non-physicians (e.g., psychologists, psychotherapists, and nurses who are experienced with substance effects). However, the legal responsibility for treatment lies fully in the hands of the treating physician. Most providers are psychiatrists. Other medical specializations or sub-specializations include anesthesia, pain medicine, internal medicine, general medicine, palliative care, neurology, psychosomatics, addiction medicine, pharmacology, and others. A physician who provides PAT may use the substance in a patient who is in his long-term care during one or a few visits per year. Typically, a psychiatrist conducts psychotherapy and pharmacotherapy in a patient and may then add single PAT sessions throughout this treatment. Alternatively, a physician or clinic may offer PAT to patients who are referred solely or mainly for PAT, and the patients would then return to their primary care physicians after PAT. Psychedelic-assisted therapy typically includes one or several preparatory visits, one or several treatment visits, and one or several integration visits, depending on the setting, patient, and physician. As a minimal legal requirement, the preparatory visits include information about potential (adverse) effects of the substance, and written informed consent always needs to be obtained before applying for authorization. Treatment sessions can be conducted in a single-patient or group therapy setting. Single-patient sessions are typically conducted by one session monitor/therapist only, whereas group sessions are conducted by several therapists. There is no minimally required number of therapists for either single or group treatments. Depending on the disorder and setting, the number of preparatory, treatment, and follow-up visits may vary. Legally and as outlined in the next paragraph, the term PAT as used herein and in the Swiss PAT program and law does not necessarily include psychotherapy. Psychedelic-assisted therapy refers only to the (safe) use of the controlled substance to treat a medical condition. Thus, psychotherapy is not a prerequisite to be combined with PAT, although it is typically used before and after PAT, particularly in the treatment of psychiatric patients. The extent to which psychotherapy is used and the type of therapy that is employed vary considerably between PAT providers, settings, and treatment indications within the Swiss limited medical use program. Thus, the term “program” as used herein refers to the legal possibility of using PAT and characteristics of its use within Switzerland and is not a guideline or a fully regulated mandatory procedure.

### 3.3 Legal situation and requirements for PAT authorizations

The FOPH issues authorizations for the limited medical use of otherwise banned controlled substances, based on Article 8, paragraph 5, of the Swiss Narcotics Act (SR 812.121, Oct 3, 1951, BetmG). Such exceptional limited medical use permits may be granted, based on the following requirements that need to be met cumulatively: the patient suffers from a mostly incurable disease, the patient's suffering could potentially be alleviated by the prohibited substance, and there are no alternative treatments, or such treatments have already extensively been used unsuccessfully. An authorization is valid for one substance, one patient, and for a period of 12 months. A report on the individual outcome is required at the end of treatment. Serious adverse events need to be reported to the FOPH within seven working days. Switching to another substance is possible, but in this case a new authorization is issued. Extensions of authorizations are possible. The dose and treatment frequency are not regulated and are determined by the physician who conducts PAT, based on individual patients' assessments. Patients who are treated with PAT need to reside in Switzerland, and adequate follow-up after PAT needs to be guaranteed. With the application for an individual authorization for PAT to the FOPH, the patient's written informed consent also needs to be submitted. In the paper application, physicians must provide detailed (medical) information on the patient, the source of the controlled substance (pharmacy), and the financing. With their first application, physicians also need to document their education and continuous training in PAT, their membership in a professional society, and their professional exchange with experienced physicians in the field.

### 3.4 Treatment costs

The full cost of a single day of PAT is estimated to be CHF 3′000.00–4′000.00, but currently mostly lower amounts are charged to the patients (CHF 800.00–2′000.00). The cost for the substance is CHF 100.00–450.00, depending on the substance and dose that are used. Psychedelic-assisted therapy is currently not (fully) reimbursed by mandatory health insurance and is typically paid by the patients themselves. Sometimes, costs are covered by foundations or social assistance. Health insurance covers regular visits and/or psychotherapy, covering some of the preparation and follow-up cost. Substance and supervision on treatment days is not reimbursed and needs to be paid out of pocket by the patient or is written off by the clinic or practice. Most practices and clinics provide PAT as outpatient treatment, but some clinics offer one or several PAT sessions within an inpatient stay that lasts several days or weeks and includes other treatments. Such inpatient treatment costs are then typically covered by private health insurance or basic health insurance with an additional out-of-pocket co-payment by the patient. In many cases, clinics and/or physicians currently offer PAT to their patients at rates that do not fully cover the costs. There are different options that are being discussed to cover costs of PAT in the near future, including coverage by private health insurance or including PAT in obligations to provide benefits under compulsory healthcare insurance. For example, the provision of 15 h of conventional psychotherapy could be substituted by the coverage of PAT.

### 3.5 Professional societies

Specialized professional societies are a regulatory requirement and play a key role in the implementation of PAT. The Swiss Medical Society for Psycholytic/Psychedelic Therapy (Schweizerische Ärztegesellschaft für Psycholytische Therapie [SÄPT]) was founded in 1985 and primarily represents Swiss-German physicians who are active in PAT. Additional Swiss societies were founded more recently to represent the different language regions in Switzerland. The Swiss Society of Psychedelic Medicine (Société Suisse de Médicine Psychédélique [SSMP]) was founded in 2023 with the objective of congregating healthcare professionals who use PAT and is active within the French-speaking part of Switzerland.

The Association Professionelle Suisse–Psychédéliques en Thérapie (ASPT) is another professional association in the French-speaking part of Switzerland that is affiliated with the University Hospital Geneva. The Alaya Foundation is the professional society in the Italian-speaking southern part of Switzerland, founded in 2012. The different societies organize the professional formation of PAT, continuous medical education, super- and intervision, and local and joint conferences. The professional societies also provide ombudsmen to patients who have complaints about PAT, partly in collaboration with, for example, the Swiss association of patients who have received PAT (Psychédelos). This service should provide an ethical and secure framework for patients, ensuring conflict mediation, transparency, and impartiality. The means used are the availability of an independent ombudsman to handle complex cases, listening to and analyzing complaints, and convening joint meetings with patients and psychiatrists.

The different Swiss societies, institutional providers, research institutions, and medication providers are loosely organized together in the Swiss Interest Group PAT (IG-PAT). The IG-PAT meets regularly to address issues of common interest and plan joint meetings of societies, conferences, and meetings with regulators. Joint treatment recommendations for PAT were published by the IG-PAT, including all specialized professional societies (Aicher et al., 2024a). Other publications include patient case reports (Mueller et al., 2025; Muller et al., 2025), case series (Calder et al., 2024; Schmid et al., 2021), descriptions of PAT in Switzerland (Aicher et al., 2024b), professional training (Aicher et al., 2025), and reviews of evidence (Rougemont-Bücking et al., 2024).

The SÄPT homepage (www.saept.ch) functions as a host for the IG-PAT and also provides information on dosing, drug interactions with psychedelics, and quality control questionnaires (treatment feedback and registry) that are pertinent for all Swiss PAT societies.

There are other societies with an interest or role in PAT. In addition to professional societies that are fully specialized in PAT and open to all medical specialties, the Swiss Society of Psychiatry and Psychotherapy has also issued a position paper (Brühl et al., 2023) and guidelines on PAT in psychiatry. The Awareness Lectures on Psychedelic Science (ALPS) foundation is a Swiss student organization that mainly organizes annual conferences on PAT with the goal of educating professionals and the public on evidence-based PAT. The foundation also organizes a Summer School, including lectures, workshops, and social events. Furthermore, there are several regionally active student organizations with similar goals.

### 3.6 Psychedelic-assisted therapy education and continuous formation

Professional societies organize PAT training programs that are recognized by the FOPH as a specialized formation in PAT for physicians who apply for PAT authorization. The SÄPT offers a comprehensive 3-year training program that currently includes 24 professionals every 3 years (Aicher et al., 2025). The SSMP also offers PAT training that lasts longer than 6 months, including five in-person training days, several video meetings, and ketamine self-experience. The ASPT offers short 2- to 3-day training in PAT and introductory lectures for medical school students. All organizations offer regionally based inter- and supervision among professional colleagues and continuous education programs and workshops and participate in joint conferences. Currently, there are efforts to implement a certified proficiency certificate for physicians to practice PAT. Psychedelic-assisted therapy is currently practiced in Switzerland within different medical specializations and is not legally limited to specific indications or medical specialties. The FOPH requires proof of training by physicians who apply for authorizations. However, the formation requirements are currently not strictly regulated or fully established and differ between providers. A key aspect of qualification for the FOPH is the documentation of professional networking by means of active membership and integration in a professional society and inter- and supervision activity with professional partners.

### 3.7 Personal experience

Medically indicated self-therapy can be authorized within the Swiss limited use program, but “personal experience” to gain insight and for educational purposes is not permitted by current law. However, persons who participate in the advanced training program that is offered by the SÄPT (years 2023–2028) are eligible for an academic trial (NCT05570708) that investigates the risks and benefits of personal psychedelic experiences for therapists, including administrations of MDMA, psilocybin, and LSD, thus allowing for personal experience in a regulated and legally approved research context. In the SSMP training, ketamine is used for self-experience purposes.

### 3.8 Provider and patient registries

The FOPH and University Hospital Basel (UHB), in collaboration with the IG-PAT, collect information on providers and patients, including treatment outcomes and adverse effects, in different registries. The FOPH registers all providers and patients, including mandatory end-of-treatment outcome reports that are submitted to the FOPH by the authorized physicians. This registry tracks the number of authorizations, number and specialization of physicians who offer PAT, substances that are used, and number, sex, age, and diagnoses of patients who are treated. This registry is maintained by the FOPH and includes all authorized physicians and all patients for whom an authorization is issued. A list of most physicians who offer PAT in Switzerland is provided by the IG-PAT on the SÄPT homepage (www.saept.ch). The list of physicians who generally offer PAT aims to provide information on PAT to patients and primary care providers, thereby attempting to offer fair access to treatment where demand currently exceeds supply. The UHB, in collaboration with the IG-PAT, maintains an additional “outcome” registry, which collects some information on the indication for PAT, the way sessions are conducted (group *vs*. individual treatment, use of concomitant psychopharmacological medication), treatment outcome, and adverse effects data. The data are collected from authorized physicians using a questionnaire for each patient that is completed by the physician at the end of treatment (normally 12 months) with one substance. The questionnaire is available in German, French, Italian, and English via IG-PAT on the SÄPT homepage. The provision of outcome data for each patient to the FOPH is mandatory, whereas the contribution to outcome data of the UHB registry is currently voluntary and does not include all patients and treatments.

### 3.9 Indications for PAT

Table 1 shows the number of primary diagnoses (723) among 723 patients who were treated with PAT in Switzerland per substance in 2024. Most patients were treated for depressive disorders (MDD, TRD, etc.), followed by PTSD (including complex PTSD), anxiety disorders (including GAD), and substance use disorder (SUD). Psilocybin was mostly used in patients with depressive disorders, followed by PTSD, SUD, and anxiety disorders. MDMA was mostly used in patients with depressive disorders, followed by PTSD, anxiety disorders, and SUD. LSD was mostly used in patients with depressive disorders, followed anxiety disorders, PTSD, and SUD.

**Table 1:** Primary diagnoses of patients treated within the Swiss limited medical use program in a total of 723 patients treated in the year 2024.

Primary diagnosis	Psilocybin	MDMA	LSD
Depressive disorders	233	126	77
PTSD	33	72	8
Anxiety disorders	22	19	17
Substance use disorder	24	6	5
Cluster headache or migraine	10	0	6
OCD	3	5	3
Bipolar disorder	7	1	2
Eating disorder	4	3	2
Personality disorder	0	2	1
Psychosomatic disorders (unspecified)	2	1	0
Other	10	10	9

*PTSD*: posttraumatic stress disorder; *OCD*: obsessive-compulsive disorder; *MDMA*: 3,4-methylenedioxymethamphetamine; *LSD*: lysergic acid diethylamide.

### 3.10 Outcome and adverse effects

A first study did not collect therapeutic outcome information and only described patient characteristics (2014–2018), treatment indications, and acute responses. The study observed similar acute subjective effects of LSD and MDMA in patients in PAT compared with healthy research participants (Schmid et al., 2021).

Recently, the UHB initiated a registry and quality assessment program, including therapeutic outcome information, based on feedback that is provided by the therapist. For the present report, only a limited number of feedback reports were available for analysis (LSD = 22, MDMA = 59, and psilocybin = 21). The median (range) doses that were mostly used were 150 μg (100–200 μg) for LSD, 125 mg (75–200 mg) for MDMA, and 25 mg (10–40 mg) for psilocybin. Group therapy was more common than single therapy. The median number of treatments with a substance per patient was three (range = 1–6) within 12 months. Investigators/therapists provided an overall median rating of acute effects (“any acute effects”) of 8 on a scale from 0 to 10. The intensity of “acute good drug effects” was rated as 6–8, and the intensity of “bad drug effects” was 3–5.5 (median ratings per substance for the whole authorization period, with scales ranging from 0 to 10). Median illness severity ratings on the Clinical Global Impression Scale (CGI) by physicians/therapists at the beginning of treatment were 6 on a scale from 1 to 7, indicating high disorder severity. Median CGI global improvement ratings were 1–2, consistent with much improvement by the end of the treatment duration of mostly 12 months. The median CGI therapeutic efficacy was rated as 2 on a scale from 1 to 4, indicating clear improvement. Adverse effects mainly included acute headache, nausea, palpitations, sleep disorders, anxiety, psychological destabilization, and flashbacks. Suicidal thoughts were rare, and no suicidality was reported.

## 4 Conclusion

The Swiss PAT framework has been set up mainly from the bottom up, slowly developing more than 10 years ago while learning by doing by treatment providers in collaboration with a pragmatic regulator.

Swiss law never prohibited the medical use of controlled substances and included the exceptional use of otherwise controlled narcotics in the law since 1951. While narcotics laws, regulations, and implementations were and still are more restrictive with regard to PAT in many countries since the 1970s, LSD and MDMA were used in PAT in Switzerland in the 1960–70s and in 1988–1993 (Gasser, 1994, 1996). Clinical studies with MDMA, psilocybin, and LSD were conducted in Switzerland starting in the 1990s (Gasser et al., 2014; Hasler et al., 1997; Oehen et al., 2013; Vollenweider et al., 1998). The current Swiss PAT program, which started in 2014, was also supported and accelerated by clinical research on the clinical pharmacology of MDMA, LSD, and psilocybin, supporting further information on dose selection (Holze et al., 2022, 2024; Liechti and Holze, 2022; Studerus et al., 2021; Vizeli et al., 2024), drug-drug interactions (Becker et al., 2022; Becker et al., 2025), safety (Schmid et al., 2015; Straumann et al., 2024; Vizeli and Liechti, 2017), and efficacy in patients (Gasser et al., 2014; Holze et al., 2023, 2024; Mueller et al., 2025; Oehen et al., 2013). The substances also became available for PAT via the supply set-up for clinical studies. Thus, Switzerland was very active in psychedelic research and PAT practice, and there was a foundation to build on, which facilitated set-up of the program. Nevertheless, it took 10 years to reach the current, albeit still very small, program size, with approximately 100 physicians who treat less than 1000 patients per year.

The Swiss PAT program was initiated several years before the more recent limited access programs in Canada and Australia, starting at a time of still very limited treatment efficacy and safety data and before the broader interest in PAT that has been seen in recent years. There are specific features and notable differences between the programs. For example, only the Swiss PAT program allows the use of LSD. In Canada and Australia, only MDMA and psilocybin are available, and they are primarily used for the treatment of PTSD and depressive disorder, respectively. The Swiss PAT program is not limited to certain indications. If evidence of efficacy becomes available for a new indication, then treatment for this indication may rapidly be approved. Treatment is also available for indications with very limited evidence of efficacy. For example, MDMA is most often used in patients with depressive disorder rather than in patients with PTSD in the Swiss PAT program, while evidence of efficacy in depressive disorders has only very recently been generated in clinical studies (Kvam et al., 2022). The Australian program is restricted to psychiatrists, whereas medical doctors with licenses to practice with board certifications other than psychiatry may offer PAT in Switzerland. In contrast to the Australian program, physicians can treat patients alone without the need for a dyad of health professionals, and there is no need for ethics committee approval before treating a patient. Thus, the Swiss program may be seen as less restricted and less regulated in some aspects. This is also clearly different from the stricter requirements to document efficacy for a drug to get market access, reflecting the different regulations. There is a risk that PAT is offered for indications for which it may be shown to be ineffective once more research is available.

In the present report, we sought to characterize the Swiss limited use program for PAT to potentially inform patients and regulators and mostly potential treatment providers in other countries. Such an approach has limitations. The Swiss program has unique features, and regulations may vary in other countries. Additionally, information on the present program was limited partly because of legal reasons. For example, for 2024, complete information was available to this report's main authors on the number of physicians who hold authorizations for PAT, the number of patients with an authorization for PAT, and patient sex, age, and diagnosis. However, outcome data were only available for a limited number of patients. This is attributable to the fact that the presently used “outcome” registry set-up by professionals who are involved in PAT is currently not mandatory. In contrast, the regulator's registry is mandatory, but outcome data have never been reported, and it is inaccessible to professionals. The goal is to jointly assess and evaluate treatment outcomes in a standardized manner and in all patients in the future, which would allow to learn more from the relatively high number of treated patients. Outcome data would ideally be collected comprehensively and similarly across different countries with limited access programs. Collecting and providing more detailed information on patients who are treated with PAT within limited access programs would be important to oversee treatment trends, gain more clinical experience, and ensure quality and safety. More detailed information would be of particular interest to those who aim to set up comparable limited access programs in other countries.

The first products for PAT will likely not reach the market before 2028. Therefore, restricted access programs would be needed as an option to assess psychedelics as medical treatments. Licensed treatments are likely to be first available only in the United States or only in a few countries and with potential limitations, and limited access programs are needed beyond the availability of marketed medications. Limited access programs are also useful for gaining experienced with this new treatment form and training physicians. Additionally, licensed products will likely be more expensive than products that are currently provided in limited use programs. Limited medical use programs may likely continue to exist, even if and when marketed products become available and are likely more cost-effective (Gisby and Wolswijk, 2025). However, we are unaware of any other established early access programs in Europe. In the European Union, early access schemes have various names, such as “Compassionate Use Programs,” “Named Patient Programs,” or “Special Access Programs” (Balasubramanian et al., 2016). These programs allow patients who are ineligible for a trial to be treated with the study medication outside an ongoing clinical trial, often involving the clinical trial sponsor, typically a pharmaceutical company. This type of early access program is legally and practically distinct from the presently described limited medical use program in Switzerland, which does not involve a pharmaceutical company or trial sponsors. The EU programs vary widely across countries in terms of requirements, scope, and implementation. The European Medicines Agency provides non-binding recommendations on compassionate use under Article 83 of Regulation (EC) No. 726/2004, and these programs are ultimately managed at the national level, resulting in significant fragmentation across Europe. The Czech Republic recently passed legislation that will allow the use of psilocybin in mental health conditions. Other national professional societies have started engaging with their governments and/or regulators to explore possibilities of some form of compassionate use or restricted access to psychedelics. However, there may be resistance from regulators to embrace early access because they might argue that psychedelics and MDMA are considered medicinal products and, as such, need to be developed following regular routes to European Medicines Agency or national registration. As part of the ongoing revision of the EU pharmaceutical legislation, which is expected to go into force in 2026, the concept of regulatory sandboxes is also being introduced in the EU. Still in its early stages and not yet implemented, regulatory sandboxes are being explored as a new policy tool to facilitate innovation in medicines regulation, allowing the testing of new approaches within a controlled environment. At the EU level, this could offer a valuable opportunity to advance access to psychedelic therapies and help overcome the current fragmentation of compassionate use frameworks by piloting a common model that could be adopted by interested member states, such as in the area of psychedelics for end-of-life care or TRD, ketamine for TRD, or MDMA for PTSD.

In some countries, clinical naturalistic trials may be an alternative to special access programs and may provide more comprehensive and standardized data collection while likely having a narrower scope (covering only a few indications) and entailing higher regulatory requirements and workload for the participating physicians.

In summary, we described the framework and real-world characteristics of Swiss PAT activities, one of the few PAT programs worldwide. Such information could be of interest to PAT stakeholders, including professionals, patients, and regulatory bodies that are considering setting up restricted access programs.

## Author contributions

MEL analyzed the data and wrote the manuscript. YS, FM, PG, HDA, and TH revised the manuscript.

## Role of the funding source

This work was supported by internal funds from the 10.13039/100016015University Hospital Basel.

## Declaration of competing interest

The authors declare the following financial interests/personal relationships which may be considered as potential competing interests: Matthias Liechti reports a relationship with Mind Medicine Inc. That includes: consulting or advisory, equity or stocks, and funding grants. If there are other authors, they declare that they have no known competing financial interests or personal relationships that could have appeared to influence the work reported in this paper.
